# Safety of chronic high-dose calcium channel blockers exposure in children with pulmonary arterial hypertension

**DOI:** 10.3389/fcvm.2022.918735

**Published:** 2022-09-07

**Authors:** Yan Wu, Fu-Hua Peng, Xin Gao, Xin-Xin Yan, FengWen Zhang, Jiang-Shan Tan, Song Hu, Lu Hua

**Affiliations:** ^1^Center for Respiratory and Pulmonary Vascular Diseases, Department of Cardiology, National Center for Cardiovascular Diseases, National Clinical Research Center of Cardiovascular Diseases, Fuwai Hospital, Chinese Academy of Medical Sciences and Peking Union Medical College, Beijing, China; ^2^Department of Structural Heart Disease, National Center for Cardiovascular Disease, Fuwai Hospital, Chinese Academy of Medical Sciences and Peking Union Medical College, Beijing, China

**Keywords:** pediatric, pulmonary arterial hypertension, calcium channel blocker, diltiazem, high-dose, toxicity

## Abstract

**Background:**

Chronic calcium channel blockers (CCBs) are indicated in children with idiopathic/heritable pulmonary arterial hypertension (IPAH/HPAH) and positive response to acute vasodilator challenge. However, minimal safety data are available on the long-term high-dose exposure to CCBs in this population.

**Methods:**

Patients aged 3 months to 18 years who were diagnosed with IPAH/HPAH and treated with CCB in the past 15 years were retrospectively reviewed. The maximum tolerated dose and the long-term safety of high-dose CCBs on the cardiovascular and noncardiovascular systems were assessed.

**Results:**

Thirty-two eligible children were enrolled in the study, with a median age of 9 (6–11) years old. Thirty-one patients were treated with diltiazem after diagnosis. The median maximum tolerated dose was 12.9 (9.8–16.8) mg/kg/day. Children younger than 7 years used higher doses than children in the older age group, 16.4 (10.5–28.5) mg/kg/day vs. 12.7 (6.6–14.4) mg/kg/day, *P* < 0.05. Patients were followed up for a median period of 6.2 (2.6–10.8) years. One patient died from a traffic accident, and others showed a stable or improved WHO functional class status. Thirteen (40.6%) and 10 (31.3%) patients developed arrhythmias and hypotension. Nine (28.1%) patients had sinus bradycardia, five (21.9%) had first-degree or second-degree type II atrial-ventricular blocks, and two (6.3%) had second-degree type II atrial-ventricular blocks. Most of these arrhythmias were transient and relieved after CCB dose adjustment. The most reported noncardiovascular adverse effect was gingival hyperplasia (13, 40.6%), accompanied by different degrees of dental dysplasia. No liver or kidney dysfunction was reported.

**Conclusion:**

Diltiazem was used in a very high dose for eligible children with IPAH/HPAH. The toxicity of long-term CCB use on the cardiovascular system is mild and controllable. Clinicians should also monitor the noncardiovascular adverse effects associated with drug therapy.

## Introduction

Pediatric idiopathic/heritable pulmonary arterial hypertension (IPAH/HPAH) is a rare disease characterized by increased pulmonary vascular resistance (PVR) and pressure, leading to right ventricular failure and death (1). The annual incidence for IPAH from the Netherlands registry was 0.7 cases per million children (2). If left untreated, the median survival is only 10 months (3). Patients are classified as responders and nonresponders according to the acute pulmonary vascular response to vasodilator challenge during a right heart catheterization. Responders can benefit from high-dose calcium channel blockers (CCBs) with a 5-year survival of 97% (4). At the same time, nonresponders can only be treated with targeted therapies with a much worse 5-year survival (48%) (5).

CCBs such as diltiazem, amlodipine, and nifedipine can be used to treat responders in IPAH (6–10). These drugs should be started at a low dose and progressively titrated to the maximum amount based on each patient, considering patients' cardiac function, heart rate, and blood pressure (4, 5, 7, 11, 12). Traditionally, the dose in this clinical setting is much higher than that typically suggested for other pediatric indications. However, little is known about the maximum tolerated dose of CCB in this patient population and its long-term safety over high-dose exposure.

In this study, we retrospectively reviewed children with IPAH/HPAH who were identified responders in the last 15 years at Fuwai Hospital. The study aimed to determine the maximum CCB dose in these patients and assess the long-term cardiovascular and noncardiovascular safety of CCB therapy.

## Methods

### Study design and participants

The study population was patients aged 3 months to 18 years, diagnosed with IPAH/HPAH, and who responded to acute vasodilator challenge. All consecutive patients who visited Fuwai Hospital between January 2006 and March 2021 were retrospectively reviewed on the hospital's electronic medical platform. The patients' clinical characteristics and hemodynamic data at the time of diagnosis and the data during follow-up were collected and abstracted by trained study personnel using a standardized electronic case record database. The blood sugar, liver or renal function was monitored by biochemical test every time patients were followed up. The detailed scheme for the CCB dose titration and its adverse effects was obtained from medical records. Follow-up data were recorded annually through outpatient visits, hospitalizations, or by telephone. The last date of follow-up was September 2021.

Ethical approval was obtained from the Fuwai Hospital Research Ethics Committee (No. 2021-1484). Written consent was obtained from the guardian of each patient.

### Patients' diagnosis and CCB treatment

The diagnosis of IPAH/HPAH conformed to the Third World Symposium on Pulmonary Hypertension (2003). The hemodynamic criteria were right heart catheterization (RHC), demonstrating a mean pulmonary arterial pressure (mPAP) ≥25 mm Hg at rest, mean pulmonary artery wedge pressure (mPAWP) ≤15 mm Hg, and pulmonary vascular resistance index (PVRi) ≥3 WU·m^2^ (13).

Acute vasodilator response (AVR) testing was performed in all patients during RHC. A positive AVR was defined according to the Sitbon criteria: a decrease in mPAP after vasodilator challenge of at least 10 mm Hg to a value of <40 mm Hg without change or an increase in cardiac output relative to baseline value (5).

Responders were treated with CCB, with or without a combination of targeted therapy according to the physician's judgment. Drugs administered were diltiazem, amlodipine, or nifedipine. The choice depended on the patient's heart rate (HR), systemic arterial pressure (SAP), and adherence. The diltiazem dose adjustment scheme was as follows: starting with an initial dose of 1.5–2.0 mg/kg per day in three divided doses and increasing the dose every 2–4 weeks to the maximum tolerated dose. Amlodipine and nifedipine were alternatives when the resting HR was < 70 beats/min (bpm).

### Statistical analysis

As appropriate, data are presented as mean ± SD, median (interquartile interval), and number (%) of patients. Wilcoxon's rank-sum test was used to compare differences in CCB dose between different age groups. *P* < 0.05 was considered statistically significant. All statistical analyses were performed using SPSS (SPSS Inc., Chicago, IL, USA).

## Results

### Study group

One hundred and fifty children met the IPAH/HPAH criteria, and all had vasoreactivity tested during RHC. Thirty-two children (21%) were identified as responders to acute vasodilator challenge and were included in the study. These patients were treated with CCB immediately after diagnosis. The baseline clinical characteristics of the patients are listed in Table 1.

**Table 1:** Baseline clinical characteristics of patients.

**Characteristics**	
**Demographics**	
Age (years)	9 (6–11)
Height (cm)	134 ± 23
Weight (kg)	30 ± 12
BSA	1.16 ± 0.25
Female, *n* (%)	22 (68.8)
HR (beats/min)	95 ± 16
mSAP (mmHg)	75 ± 10
**WHO FC, *n* (%)**	
FC I	1 (3.1)
FC II	22 (68.8)
FC III	9 (28.1)
**Biochemical test**	
NT-proBNP (pg/ml)	306 (136–1,495)
ALT (umol/l)	21 ± 12
AST (umol/l)	30 ± 12
UA (umol/l)	347 ± 83
Cr (umol/l)	47.7 ± 12.7
**Echocardiogram**	
LVEDD (mm)	34 ± 7
RV (mm)	23 ± 7
sPAP (mmHg)	66 ± 19
TAPSE (mm)	17 ± 3
**Hemodynamics from RHC**	
mPAP (mmHg)	49 ± 10
PVRi (WU/m^2^)	11.9 ± 4.3
CI (L/min/m^2^)	3.6 ± 1.2
**Maximum diltiazem dose (mg/day)**	360 (240–420)
**Maximum diltiazem dose (mg/kg/day)**	12.9 (9.8–16.8)
> 7 years old (*n* = 17)	12.7 (6.6–14.4)
^*^ <=7 years old (*n* = 14)	16.4 (10.5–28.5)

Values are mean ± SD; median (interquartile interval); and n (%).

* P < 0.05 when compared with doses in children older than 7 years.

BSA, body surface area; mSAP, mean systemic arterial pressure; HR, heart rate; FC, functional class; NT-proBNP, N-terminal pro-B-type natriuretic peptide; ALT, Alanine transaminase; AST, aspartate aminotransferase; UA, urine acid; Cr, creatinine; LVEDD, left ventricular end-diastolic dimension; RV, right ventricular; sPAP, systolic pulmonary arterial hypertension; TAPSE, tricuspid annulus plane systolic excursion; mPAP, mean pulmonary artery pressure; PVRi, pulmonary vascular resistance index; CI, cardiac index.

The median age of PAH diagnosis was 9 (6–11) years old, 22 (68.8%) were females, and 23 (71.9%) had the World Health Organization (WHO) Functional Class I-II. The level of N-terminal pro-B-type natriuretic peptide (NT-proBNP) elevated to 306 (136–1,495) pg/ml at diagnosis (14). The mean baseline SAP and HR were 75 ± 10 mmHg and 95 ± 16 bpm. All children had normal liver and renal functions before CCB initiation. The echocardiogram observed a marked elevation in systolic pulmonary arterial pressure (66 ± 19 mmHg) and a low tricuspid annulus plane systolic excursion (17 ± 3 mm). mPAP and PVRi were 49 ± 10 mmHg and 11.9 ± 4.3 WU·m^2^ from the diagnostic RHC.

### Long-term oral high-dose CCB for patients

All children started oral CCBs after diagnosis. Detailed information for CCB dose is shown in Tables 1, 2. Thirty-one patients were treated with diltiazem and one with amlodipine due to a baseline HR of < 70 bmp. At the end of the follow-up, 28 patients were still taking diltiazem and two with amlodipine (including one patient taking diltiazem and amlodipine combination therapy). One patient did not respond to long-term CCB treatment and was switched to PAH-targeted therapy.

**Table 2:** Detailed clinical information of each patient.

**ID**	**Age**	follow-up^*^	**CCB**	**Maximum dosage**	**Side-effects**	**mSAP (mmHg)**		**HR (beat/min)**		**WHO FC**	
	**(years)**	**(years)**	**therapy**	**(mg/kg/day)/(mg/d)**		**baseline**	**follow-up**	**baseline**	**follow-up**	**baseline**	**follow-up**
1	0.7	2.6	diltiazem	11.3/135	II-I AV-block	70	60	105	88	2	2
2	2	1.4	diltiazem	17.1/240	gingival hyperplasia, increase in body hair	73	60	102	65	3	2
3	3	13.3	diltiazem	32.0/480	sinus bradycardia, gingival hyperplasia, increase in body hair	85	78	98	65	2	1
4	3	5.1	diltiazem	34.3/480	sinus bradycardia, gingival hyperplasia	84	65	120	94	2	2
5	3	2.7	diltiazem	7.5/120	sinus bradycardia, II-I AV-block, hypotension	66	58	117	75	3	1
6	3	1.7	diltiazem	28.0/420	sinus bradycardia, gingival hyperplasia, increase in body hair	67	55	90	56	3	2
7	5	12.5	diltiazem	30.0/600	hypotension, gingival hyperplasia, constipation	80	65	102	66	3	1
8	5	2.2	diltiazem	10/210	none	61	52	142	114	2	1
9	6	5	diltiazem	12.3/270	none	86	72	71	61	2	2
10	6	6.1	diltiazem	7.8/180	sinus bradycardia, atrial tachycardia, hypotension	77	68	77	112	3	1
11	6	11.1	diltiazem	19.6/450	sinus bradycardia, I degree AV-block	75	57	102	59	2	2
12	7	14.4	diltiazem	17.5/420	constipation	70	58	96	70	2	2
13	7	6.3	diltiazem	15.7/360	none	74	68	87	86	2	2
14	7	3.2	diltiazem	10.6/255	hypotension, gingival hyperplasia	73	60	95	80	2	1
15	8	13.2	diltiazem	16.2/420	II-I AV-block	68	59	110	75	1	1
16	8	14.4	diltiazem	16.8/420	gingival hyperplasia	50	50	100	75	2	1
17	9	2.6	amlodipine	0.38/5	sinus bradycardia, hypotension	81	71	79	70	3	1
18	9	13.5	diltiazem	12.9/360	hypotension, gingival hyperplasia	61	58	88	70	2	1
19	9	6.5	diltiazem	13.3/360	gingival hyperplasia, increase in body hair	102	63	99	74	2	2
20	10	2.6	diltiazem	13.0/390	none	74	66	77	67	2	2
21	10	5.7	diltiazem	15.5/480	none	74	64	63	75	2	2
22	10	9.9	diltiazem	10.9/360	gingival hyperplasia	79	64	110	69	3	1
23	11	3.5	diltiazem	9.8/315	hypotension, rash	72	63	78	63	2	1
24	11	6.9	diltiazem	13.3/465	none	79	69	86	74	2	2
25	11	2.4	diltiazem	16.4/525	gingival hyperplasia, hypotension	68	60	108	77	3	2
26	12	13.7	diltiazem	12.7/420	gingival hyperplasia, increase in body hair	75	70	84	70	2	1
27	12	8.6	diltiazem and amlodipine	10.6 and 0.16/330/2.5	sinus bradycardia, gingival hyperplasia	72	59	98	60	2	2
28	15	0.6	diltiazem	4.9/180	none	107	death	87	death	3	death
29	15	9.7	diltiazem	3.3/120	II-II AV-block, hypotension	90	88	98	89	2	2
30	15	1.6	diltiazem	7.1/270	Rash	70	58	108	64	2	1
31	17	9.1	diltiazem	6/240	sinus bradycardia, II-I AV-block, hypotension	84	70	89	70	2	2
32	17	9.1	diltiazem	5.5/225	II-II AV-block, atrial tachycardia, fatigue, dizziness	98	83	82	84	2	2

* The median follow-up period is 6.2 (2.6–10.8) years.

^&^the patient died from traffic accident 7 months after diagnosis.

CCBs, calcium channel blockers; AV-block, atrial-ventricular block; mSAP, mean systemic arterial pressure; HR, heart rate; FC, functional class.

The median maximum tolerated diltiazem dose was 12.9 (9.8–16.8) mg/kg per day, ranging from 3.3 to 34.3 mg/kg per day. Children younger than 7 years used a higher dose than patients in the older age group, 16.4 (10.5–28.5) mg/kg/day *vs*. 12.7 (6.6–14.4) mg/kg/day, *P* < 0.05. The six children younger than 3 years old at diagnosis were all treated with diltiazem during the entire follow-up period. The maximum tolerated diltiazem dose in these six youngest patients ranged from 7.5 mg/kg/day to 34.3 mg/kg/day.

### The effects of high-dose CCB treatment

The patients were followed for a median period of 6.2 (2.6–10.8) years. A patient died from a traffic accident, although his symptoms were markedly relieved with diltiazem. All other children had stable or improved WHO functional class and normal NT-proBNP, 14 (45.2%) in functional class I and 17 (54.8%) in functional class II (Table 2). Systolic pulmonary arterial pressure and tricuspid annulus plane systolic excursion were improved to 37 ± 9 mmHg and 21 ± 3 mm.

### Adverse effects of high-dose CCB

The adverse effects of CCB are shown in Tables 2, 3. Thirteen patients had arrhythmias: nine (28.1%) sinus bradycardia (HR < 60 bpm), five (21.9%) first-degree or second-degree type I atrial-ventricular block (AV block), and two (6.3%) second-degree type II AV block. No patient had third-degree AV block, atrial flutter, and atrial fibrillation. Hypotension occurred in 10 patients (31.3%): four complained of mild orthostatic dizziness accompanied by nausea, three complained of fatigue when exercising, and the rest were asymptomatic. The symptoms of bradyarrhythmias and hypotension were relieved after optimizing the diltiazem dose, except for the patient with second-degree type I AV block. Severe hypotension, cardiac arrest, and cardiogenic shock were not documented during the study.

**Table 3:** Long-term adverse effects of calcium channel blockers.

**Side effect of calcium channel blocker**	***n* (%)**
Sinus bradycardia	9 (28.1)
I degree/ II degree I type A-V block	5 (21.9)
II degree II type A-V block	2 (6.3)
Hypotension	10 (31.3)
Gingival hyperplasia	13 (40.6)
Increase in body hair	5 (15.6)
Others	
Rash	2 (6.3)
Constipation	2 (6.3)
Liver dysfunction	none
Renal dysfunction	none

AV-block, atrial-ventricular block.

**Figure 1:**
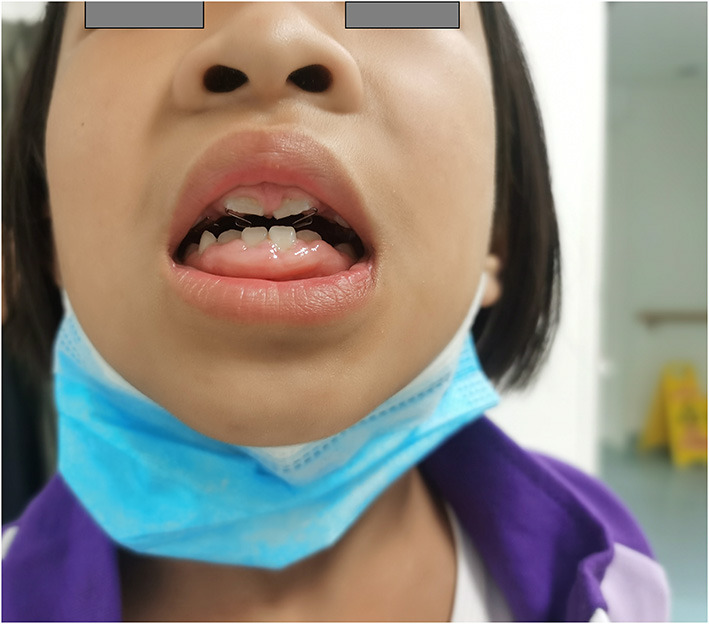
A patient with severe gingival hyperplasia. Female, 3 years old at diagnosis, treated with diltiazem at the maximum dose of 34.3 mg/kg/day, gingival hyperplasia is severe, companied with dental abnormalities.

Gingival hyperplasia was reported in 13 (40.6%) patients, accompanied by a different degree of dental dysplasia (Figure 1). The adverse effect mostly occurred 16 months after initiation of CCB and when the dose was more than 10 mg/kg per day. Other adverse effects included were increase in body hair (*n* = 5), constipation (*n* = 2), and rash (*n* = 2). Hyperglycemia and liver or kidney dysfunction were not reported.

## Discussion

In pediatric patients with IPAH/HPAH, only a minority can benefit from chronic treatment with CCB. These patients are defined as vasodilator responders. This subset of the population is very rare. In the past 15 years, we diagnosed 150 pediatric IPAH/HPAH patients in our PAH center; only 32 patients (21%) were positive responders. Similarly, in a global registry of pediatric PAH and pediatric REVEAL cohort from the United States, only 32 and 19 children were responders using the same criteria (8, 10). Most previous studies focused on the long-term effect of CCBs in pediatric patients with IPAH/HPAH. There are minimal data on the actual use of CCBs, their maximum tolerated doses, and whether chronic exposure to high-dose CCB results in severe toxicity to the cardiovascular system or other body organs. In the present study, we attempted to answer these questions by reviewing our eligible patients to characterize the safety of chronic high-dose CCBs in pediatric patients.

Initially, CCBs were studied as vasodilator agents to test pulmonary vasoreactivity (6, 15, 16). However, their use is limited by possible severe adverse clinical events, including profound hypotension and cardiogenic shock. Therefore, vasodilators that are more selective to the pulmonary arteries are used. The guidelines recommend that CCBs only be indicated in PAH patients with significant acute pulmonary vasoreactivity (17, 18). CCBs should start from a low dose and then gradually titrated upward to the maximum tolerated dose over weeks to months.

Long-term CCB responders in adult PAH patients have been treated with diltiazem at a mean daily dose of 482 ± 151 mg (range, 180 to 720 mg), nifedipine 102 ± 27 mg (range, 60 to 120 mg), or amlodipine 20 mg, without reported severe cardiovascular toxicity (5). The following CCB regimens have been recommended for pediatric patients with PAH: diltiazem at the initial dose of 1.5–2 mg/kg/day in three divided doses and the maintenance dose of 3–5 mg/kg/day in three divided doses, or nifedipine started at 0.6–0.9 mg/kg/day with a maintenance dose of 2–5 mg/kg/day (19). However, maximum tolerated doses have not been reported or recommended. On the contrary, the maximum dose of diltiazem for pediatric hypertension was said to be 6 mg/kg per day up to 360 mg per day (20).

Our study found that diltiazem was the most frequently administered CCB in our practice. The median maximum tolerated diltiazem dose was 12.9 mg/kg per day, several times higher than the recommended maintenance dose for pediatric patients with IPAH/HPAH or the maximum dose for pediatric hypertension. Younger patients seemed to tolerate higher doses, calculated by body weight, than older ones. In this study, a 3-year-old patient received the highest diltiazem dose, 34.3 mg/kg/day. Similar to the findings of other pediatric IPAH/HPAH cohorts, CCBs worked well in our patient population to improve cardiac function and survival (4, 8, 10). This may be explained because PAH patients who responded positively to acute vasodilator challenge are supposed to have almost exclusively vasoconstrictive abnormality without severe cellular narrowing of the arterioles (12). Pulmonary vasoconstriction could be relieved or reversed by high-dose CCB.

Several factors might have contributed to the high utilization of diltiazem in our practice. First, most children were documented to have a rapid HR when PAH was diagnosed, and diltiazem has a negative chronotropic effect to counteract the HR. Second, the half-life of diltiazem is shorter than that of amlodipine, which enables physicians to handle complications in case emergencies occur. Acute CCB overdose was ranked as the top sixth overall substance category associated with the most reported fatalities. Pediatric poisonings are mainly unintentional ingestions (< 6 years old) or intentional exposure (13 to 19 years old) (21). However, most literature focused on acute CCB overdose in pediatric patients. Data for chronic high-dose CCB toxicity are minimal. We found that the dose titration strategy managed by the PAH professionals displayed satisfactory safety over a long follow-up period even though most patients received higher doses of CCBs. A small proportion of patients were recorded to have bradycardia, conduction disturbances, or hypotension. However, most of these symptoms were transient and moderate and disappeared after adjusting the diltiazem dose. No emergency treatment of overdose toxicity was reported.

Theoretically, diltiazem has a negative inotropic effect and can decrease cardiac contractility.

In contrast, we observed improvement in cardiac function during the follow-up. This is also attributed to intensive pulmonary artery dilation and decreased right ventricular afterload under high-dose CCB treatment. Throughout our study, hepatic or renal function impairment and the potential adverse effect of hyperglycemia were not recorded.

Gingival hyperplasia was the most common chronic noncardiovascular adverse effect reported during follow-up, characterized by an excessive enlargement of gingival tissue. The disorder is also widely reported in adults. The prevalence rates of nifedipine- or amlodipine-induced gingival hyperplasia were 20 to 50% (22, 23) and 3.3% (24). The median onset of this adverse effect was 262 days, and more men were present with this than women (22–24). However, data in pediatrics are rare. In this study, 40.6% of children with diltiazem presented with gingival hyperplasia, mainly 16 months after starting the drug with a diltiazem dose of more than 10 mg/kg per day. Gingival hyperplasia can interfere with esthetics, chewing, speech, and psychological health, especially in children. However, management is challenging (25). Given the devastating character of PAH, changing CCB therapy is not wise or perhaps not recommended. PAH experts, stomatologists, and psychologists should work together to find the most appropriate alternative therapies for very severe cases.

## Limitations

This study suffers from limitations with the retrospective design and single-center data. Pediatric PAH centers are rare in China, especially 10 years ago. As the largest PAH center in China, we treat patients throughout the country, and our data represent wide Chinese patients. Furthermore, we enrolled all eligible children treated at our hospital for a 15-year period with continuous observation. The patients were followed for a median of more than 6 years. As a rare disease, our relatively large cohort of patients and the long duration of the study effectively avoided selective bias and ensured the representativeness of the study.

## Conclusion

The appropriate administration strategy of long-term high-dose CCBs in pediatric PAH patients is effective with mild to moderate adverse effects. Close lifelong monitoring is necessary to continuously observe the growth, development, and organ functions in this unique population.

## Data availability statement

The original contributions presented in the study are included in the article/supplementary material, further inquiries can be directed to the corresponding author.

## Ethics statement

The studies involving human participants were reviewed and approved by Research Ethics Committee of Fuwai Hospital. Written informed consent to participate in this study was provided by the participants' legal guardian/next of kin. Written informed consent was obtained from the minor(s)' legal guardian/next of kin for the publication of any potentially identifiable images or data included in this article.

## Author contributions

YW wrote the analysis plan and had the primary responsibility for writing the article. XG and LH supervised the study and data analysis. YW, XG, and LH designed the protocol. YW, F-HP, and X-XY designed the data collection forms. YW, F-HP, X-XY, XG, FZ, LH, J-ST, and SH collected the data. YW, LH, FZ, J-ST, and SH analyzed the data. YW, F-HP, X-XY, XG, FZ, and LH interpreted the data. All authors provided critical reviews of drafts and approved the final submitted version.

## Funding

This study was supported by Chinese Academy of Medical Sciences Innovation Fund for Medical Sciences (2016-I2M-1-002 and 2017-I2M-3-003), National Clinical Research Center for Cardiovascular Diseases, Fuwai Hospital, Chinese Academy of Medical Sciences (NCRC2020007), and Research Project of Clinical Toxicology from the Chinese Society of Toxicology (2021-ZX036).

## Conflict of interest

The authors declare that the research was conducted in the absence of any commercial or financial relationships that could be construed as a potential conflict of interest.

## Publisher's note

All claims expressed in this article are solely those of the authors and do not necessarily represent those of their affiliated organizations, or those of the publisher, the editors and the reviewers. Any product that may be evaluated in this article, or claim that may be made by its manufacturer, is not guaranteed or endorsed by the publisher.
